# RF Ablation of Giant Hemangiomas Inducing Acute Renal Failure: A Report of Two Cases

**DOI:** 10.1007/s00270-016-1415-1

**Published:** 2016-07-07

**Authors:** Aukje A. J. M. van Tilborg, Helena F. Dresselaars, Hester J. Scheffer, Karin Nielsen, Colin Sietses, Petrousjka M. van den Tol, Martijn R. Meijerink

**Affiliations:** 1 Departments of Radiology and Nuclear Medicine, VU University Medical Center, De Boelelaan 1117, 1081HV Amsterdam, The Netherlands; 2 Department of Nefrology, VU University Medical Center, Amsterdam, The Netherlands; 3 Department of Surgical Oncology, Gelderse Vallei Hospital, Ede, Amsterdam, The Netherlands; 4 VU University Medical Center, Amsterdam, The Netherlands

**Keywords:** Radiofrequency ablation, Giant cavernous hemangioma, Hemolysis, Acute kidney injury

## Abstract

**Objective:**

In patients that require treatment for hepatic giant cavernous hemangiomas (GCH), radiofrequency ablation (RFA) has been suggested to represent a safe and effective alternative to invasive surgery. In a recent report of bipolar RFA, using two expandable needle electrodes, was uneventfully performed in patients with large GCH (>10 cm). The objective of this report is to present two cases in which bipolar RFA of symptomatic GCH was complicated by acute kidney injury.

**Materials and methods:**

In 2015 we treated two patients for very large symptomatic GCH (15.7 and 25.0 cm) with bipolar RFA during open laparotomy.

**Results:**

In both patients the urine showed a red–brown discoloration directly after the ablation. They became anuric and presented with progressive dyspnea, tachypnea, and tachycardia, requiring hemodialysis for a period of 1 month in one case. Lab results revealed hemepigment-induced acute kidney. Both patients fully recovered and both showed a complete relief of symptoms at 3 months following the procedure.

**Conclusion:**

RFA for large GCHs can cause hemepigment-induced acute kidney injury due to massive intravascular hemolysis. The presented cases suggest that caution is warranted and advocate an upper limit regarding the volume of GCHs that can be safely ablated.

## Introduction

Hepatic hemangioma is the most frequently encountered solid benign liver tumor. Lesions greater than 5 cm have been referred to as giant cavernous hemangiomas (GCH) [1]. Although most hemangiomas are asymptomatic and are managed safely with observation alone, larger lesions may produce a variety of symptoms and signs, including pain, fullness, nausea, vomiting, and fever. For patients with invalidating symptoms the most renowned treatment remains surgical resection. Unfortunately, surgical resection is associated with morbidity up to 27 % and even a small risk of mortality [2–4]. Although mainly based on small case series and case reports, radiofrequency ablation (RFA) has shown promising results in the recent literature for the less-invasive treatment of relatively small GCH with only minor complications documented [5–13]. In a recent report four patients with symptomatic GCHs measuring >10 cm were uneventfully treated with bipolar RFA [14]. A remarkable volume reduction (58–92 %) coincided with complete (2/4 patients) or considerable (2/4 patients) symptom relief. This case report describes two patients with very large symptomatic GCHs who developed acute kidney injury (AKI) shortly after bipolar RFA, caused by massive heat-induced intravascular hemolysis.

## Materials and methods

In the following cases a commercially available generator (RF3000, Boston Scientific, USA) was combined with two expandable 7 cm bipolar needle electrodes (InCircle, RF Medical, USA). The procedures were performed using intraoperative ultrasound during open laparotomy under general anesthesia. Ablation protocol was similar to the previously described technique [14].

The local review board waived approval since (1) both the generator and needle electrodes have a CE mark for the ablation of liver tumors, (2) we have a well-documented and transparent prospective registry for all ablations, including our previous experience with RFA for symptomatic GCH, and (3) the multidisciplinary tumor board unanimously agreed on the indication. Patients gave written informed consent and all procedures were conducted according to the guidelines for good clinical practice. Follow-up at 3 months was performed by a contrast-enhanced CT or MRI scan with intravenous contrast. Volume reduction was calculated in percentages and symptom relief was objectified by a visual analogue scale (VAS score).

## Results

### Case 1

**Fig. 1:**
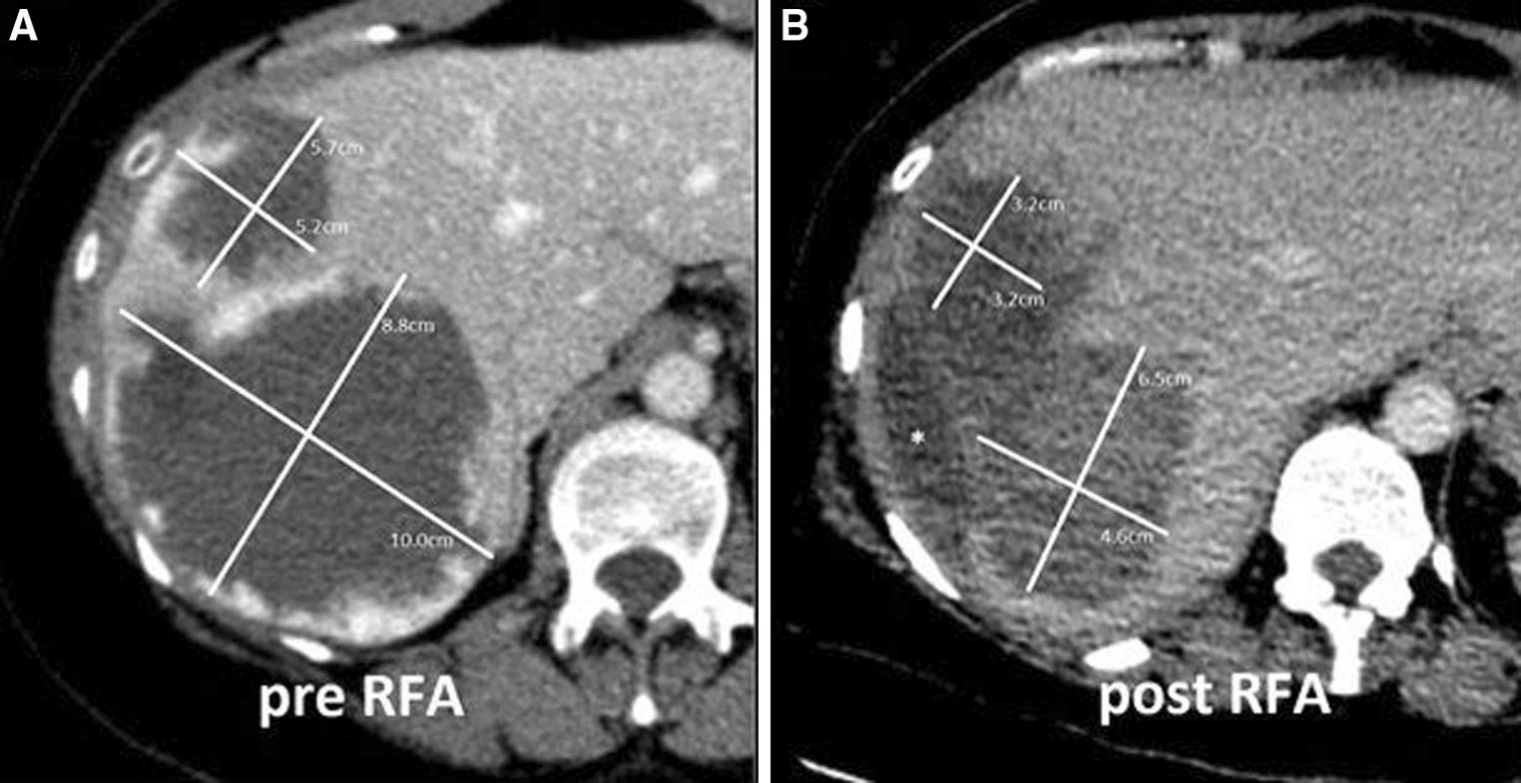
A 52-year-old female (case 1) with two large mating symptomatic GCHs in segment VII and VIII (Couinaud) of the liver (diameter of 10 and 5.7 cm) before **A** and after **B** bipolar RFA, showing a clear volume reduction. (*Fluid filled space caused by retraction of the tumor.)

A 52-year-old female, with an otherwise unremarkable history, presented with chronic fatigue and slowly progressing and invalidating pain in the upper right abdominal quadrant. Ultrasound and contrast-enhanced CT revealed the presence of two mating typical GCHs in liver segment VII and VIII (Couinaud) with a diameter of 10 cm and 5.7 cm, respectively of which the combined mass effect distorted the right hemidiaphragm (Fig. 1). The relocated and compressed right liver vein caused hypoperfusion of segment VI on the portal-venous phase CT. Surgical resection would require a portal vein embolization plus right hemihepatectomy. Both the patient and the hepatobiliary surgeon considered the risks associated with such a procedure unacceptable to treat a benign lesion. Initial arterial embolization therapy with polyvinyl alcohol particles was technically successful, but did not lead to any tumor shrinkage or symptomatic relief. Bipolar RFA was performed according to the abovementioned protocol with three overlapping ablations. Directly after the procedure the patient’s urine showed a red–brown discoloration. 1 day after the ablation the patient became anuric and subsequently developed pleural effusion with shortness of breath. On physical examination she was tachypnoic and tachycardic without any other abnormalities. Lab results showed AKI (creatinine 446 micromol/l and urea 13.4 mmol/l) and decreased hemoglobin (6.8 × 10^9/l). Peripheral blood smear showed fragmentocytes, CPK was 1726 IU/l at peak, and haptoglobin was unmeasurably low (< 0.10 g/l). Urine analysis showed 0–5 leukocytes per high-powered field and >20 erythrocytes per high-powered field (no dysmorphic erythrocytes or casts), with a total urine protein excretion of 0.35 g/day. On abdominal ultrasound the kidneys and urinary tract showed no abnormalities. Blood and urine cultures were negative. There were no indicators of preexisting systemic disease or family history of kidney disease, and the patient received no medication, besides acetaminophen. Autoimmune serology was negative as well as hepatitis B, C, and HIV serology. Because of progressive dyspnea and ongoing anuria haemodialysis through a femoral catheter was started. After 39 days kidney function recovered and dialysis was stopped and the patient was discharged from the hospital 42 days after the procedure. She had a tumor volume reduction of 56 %. The patient reported complete relief of symptoms (VAS score decreased from 6 to 0).

### Case 2

**Fig. 2:**
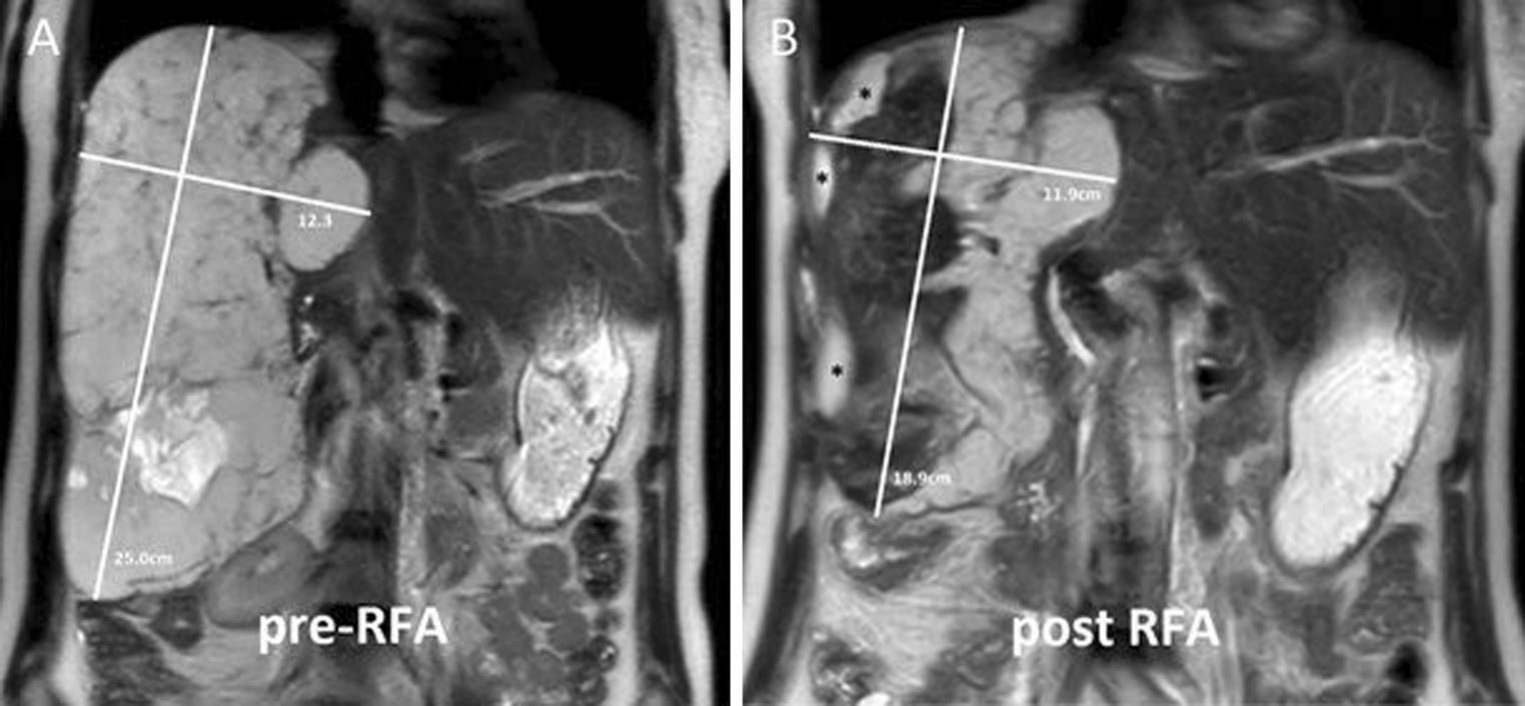
A 41-year-old female (case 2) with a colossal and enlarging GCH occupying all segments of the right liver lobe (maximum diameter 25.0 cm) before **A** and after **B** debulking bipolar RFA, showing a fair volume reduction. (*Fluid filled space caused by retraction of the tumor.)

A 41-year-old female, with no relevant medical history, suffered from progressive right upper abdominal pain and nausea for which she used morfine and pantoprazole. She had an enlarging GCH with a maximum diameter of 25 cm occupying the right liver lobe (segments V,VI,VII, and VIII) (Fig. 2). A second GCH of 5 cm was located in segment IV. The largest lesion was causing enlargement of the liver and compression of surrounding structures with an ectopic located right kidney. Surgical resection was considered too hazardous because of encasement of the inferior vena cava which would require a venous patch. The multidisciplinary team and the patient made a shared decision to perform RFA for tumor debulking. Bipolar RFA was performed according to the abovementioned protocol using a total of four overlapping ablations plus track ablations. After the procedure the patient was admitted to the intensive care, where reddish-brown urine was noted and urine dipstick turned out positive for hemoglobin. Creatinine levels increased from normal to 184 micromol/l, urea increased to 9,3 mmol/l, CPK increased to 2111 IU/l, and Hb decreased to 5.0 × 10^9/l, indicating that kidney injury was possibly caused by hemolysis. Rhabdomyolysis was too mild to be the cause of the kidney injury. Blood and urine cultures were negative. There were no indicators of preexisting systemic disease and no family history of renovascular disease. Also, no new medication was started. We started a hydration regimen and the patient received one packed cell. The next day the patient became hemodynamic and respiratory stable and was dismissed from the intensive care unit. After 8 days all lab results had normalized and the patient was dismissed from the hospital 14 days after the procedure. At 3 months follow-up both tumor shrinking (volume reduction 32 %) and symptom relief (VAS score for symptom assessment decreased from 6 to 0) were considered fair.

## Discussion

Hemepigment-induced AKI can either develop from rhabdomyolysis or intravascular hemolysis. The latter is rare and is caused by for example paroxysmal nocturnal hemoglobinuria and G6PD deficiency [15, 16]. The nearly unavoidable hemolysis after RFA, attributable to the generous blood supply of GCHs, especially for those > 10 cm, is a major drawback. Massive hemolysis can lead to various degrees of hemoglobinuria, hemolytic jaundice, anemia, and renal damage [17]. The development of AKI associated with hepatic RFA has been previously reported in the treatment of liver metastases and hepatocellular carcinoma [18–21]. Also, acute hemolysis secondary to microwave-assisted liver resection and cryoablation has been described [22, 23]. AKI as a result of RFA-induced hemolysis for giant hemangiomas or other benign liver lesions has not been reported before. Our patients developed oliguria that progressed to acute renal failure. The most likely cause of renal failure was heme-induced nefropathy caused by thermal hemolysis. The postoperative decrease in hemoglobin with fragmentocytes, the unmeasurably low haptoglobin, the postprocedural positive hemoglobin urine dipstick-test plus the reddish-brown discolored urine all support this etiology.

During RFA, the temperature of the tissue adjacent to the radiofrequency probe reaches temperatures between 50 °C and 100 °C. Red blood cells (RBCs) will undergo budding and fragmentation when exposed to a temperature of >49 °C in vitro [24, 25]. The size of the giant hemangiomas, and hence the size of the ablation zone and length of the procedure, presumably resulted in a more massive thermal hemolysis than that encountered with smaller tumors and shorter procedures.

Hemoglobin is released upon erythrocyte destruction and is filtered by the glomerulus into the urinary space. In the urinary space, hemoglobin is degraded and releases hemepigments which are toxic to the kidney. Hemepigments can cause tubular injury by (1) tubular obstruction, (2) damage due to direct proximal tubular cell injury, and (3) vasoconstriction, resulting in reduced blood flow in the outer medulla [26]. AKI that develops in the setting of hemolysis is rarely an isolated cause. Predisposing conditions, like volume depletion and, possibly, mild ischemia are often present. Volume depletion enhances both vasoconstriction and the formation of obstructing casts, and is of critical importance for the development of heme-induced AKI [27]. Our patients did not experience renal hypoperfusion caused by prolonged hypotension or hypovolemia, and renal toxic drugs like NSAID’s or cephalosporins were not administered.

Laparoscopic insufflation can also contribute to intraoperative oliguria due to increased intraabdominal pressure which can lead to decreased renal perfusion and increased renin activity [28]. In both patients the ablation was performed during open surgery, so no laparoscopic insufflation was used.

Although a percutaneous approach generally is less invasive, we preferred an open approach to avoid injury to adjacent organs, to facilitate a more aggressive ablation and to increase precision of needle advancement using intraoperative ultrasound. Moreover, the unusually large needle thickness for the ellipsoid-shaped trocar increases the risk of hemorrhage and bile duct trauma and, in its current form, seems unsuitable for percutaneous use.

In conclusion, we believe that the most likely cause of AKI in our two cases was hemolysis caused by extensive thermal ablation with a bipolar system. Laboratory monitoring after extensive or prolonged radiofrequency liver ablation procedures is recommended for early detection hemolysis. Laboratory values that should be monitored include hemoglobin, creatinine, fragmentocytes, haptoglobin, lactate dehydrogenase, CPK, and urinalysis. In addition, perioperative volume repletion should be warranted, especially if oliguria is noted and/or hemolysis is suspected. When treating large GCH with thermal ablation, the number of ablations and the duration of the ablation sequences should be reduced to the absolute minimum. For colossal tumors or multiple tumor sites a multistep approach or other treatment options should be considered to prevent hemepigment-induced AKI. The general acceptance of thermal ablation for symptomatic GCHs in routine clinical practice seems precipitated. Given the paucity of patients with an unresectable symptomatic GCH, it may prove difficult to setup larger safety studies. Outside the setting of clinical trials we recommend reticence, especially for GCHs >10 cm.
